# Variations in haematological and biochemical parameters in healthy ponies

**DOI:** 10.1186/s12917-020-02741-5

**Published:** 2021-01-19

**Authors:** Witkowska-Piłaszewicz Olga, Cywińska Anna, Michlik-Połczyńska Katarzyna, Czopowicz Michał, Strzelec Katarzyna, Biazik Anna, Parzeniecka-Jaworska Marta, Crisman Mark, Witkowski Lucjan

**Affiliations:** 1 Department of Pathology and Veterinary Diagnostics, Institute of Veterinary Medicine, Warsaw University of Life Sciences – SGGW, Nowoursynowska str. 159c, 02-787 Warsaw, Poland; 2 Faculty of Biological and Veterinary Sciences, Nicolaus Copernicus University in Toruń, Toruń, Poland; 3 Department of Internal Diseases and Veterinary Diagnostics, Faculty of Veterinary Medicine and Animal Science, Poznan University of Life Sciences, Poznań, Poland; 4 Division of Veterinary Epidemiology and Economics, Institute of Veterinary Medicine, Warsaw University of Life Sciences – SGGW, Warsaw, Poland; 5 Department of Horse Breeding and Use, University of Life Sciences in Lublin, Lublin, Poland; 6 Department of Large Animal Clinical Science, Virginia Maryland College of Veterinary Medicine, Virginia Tech, Blacksburg, USA

**Keywords:** Pony horse, Lactate, Triglycerides, Haematology, Blood biochemistry

## Abstract

**Background:**

Breed specific reference ranges for selected blood parameters are recommended for proper interpretation of blood tests, but there are only few reports dealing with ponies. The purpose of this study was to investigate if blood parameters differ among ponies’ classes and to check if general normal values for equine species are applicable to ponies.

**Results:**

All, except total protein concentration, biochemical parameter significantly (*p* < 0.05) differed among ponies’ classes. The most pronounced difference was noted in blood lactate concentrations, higher (*p* < 0.001) in the smallest ponies (class A). In all groups of ponies muscle enzymes (aspartate aminotransferase and creatine kinase) and urea were high when compared to normal values for equine species, but triglycerides and creatinine were low. Blood lactate concentration was high in comparison with normal values for horses only in class A ponies’.

**Conclusions:**

In healthy ponies, blood lactate concentration significantly differs between height classes. Normal values for equine species should not be directly applied to interpret the lactate, triglycerides, aspartate aminotransferase and creatine kinase values in ponies.

## Background

Equine breed traits manifest as unique appearance and disposition. Physiological diversity is more complex and involves blood composition, reflected by the variations in haematological and biochemical parameters. Thus, breed differences should be taken into consideration in establishing reference intervals (RI) of blood parameters and interpretation of blood tests. Moreover, the reference intervals reported in the literature may also vary due to the demographic differences such as geographic location, physical activity, age, sex, biological rhythms, etc. [1–6].

Horses are usually grouped as “hot-blooded” and “cold-blooded”. The first group generally involves the horses of Arabian ancestry, including Arabians, Quarter Horses, Standardbreds, and Thoroughbreds, whereas “cold-blooded” are basically draft type horses, including Clydesdales, Percherons, and Shires. Several differences between these two groups regarding haematological parameters have been reported, including lower haematocrit in cold-blooded and higher erythrogram values in hot-blooded horses [7]. Currently, normal values for equine species in general as well as for the most popular breeds are widely available. However, the data regarding endemic breeds and ponies are still incomplete.

In the context of haematology and blood biochemistry, ponies have been suggested to be more like “hot-blooded” than “cold-blooded” horses [8], however, other sources include ponies into “cold-blooded” [7]. Reference intervals presented in current textbooks are based primarily on Thoroughbreds and Arabians and ponies are represented by only a single breed: American Miniature Horses, for which only a few values are available [1, 7]. Consequently, clinicians must interpret the common reference intervals with caution, as they may not be directly applicable to the pony practice. Several differences are likely important in assessing general health, fitness, stress, welfare and performance or even critical for proper diagnosis in sick animals. Concentrations of L-lactate (LAC) in blood and peritoneal fluid are the most common examples, used as diagnostic and prognostic indicators of colic severity. The commonly accepted reference interval of blood lactate concentration in healthy horses is approx. 0.7 mmol/L [9, 10]. In sick horses it varies, corresponding with the diagnosis, prognosis and required treatment [9–16]. However, in ponies, data are less clear and the clinical impressions suggest that pony and miniature breeds with gastrointestinal diseases could have higher blood lactate concentration (LAC) than large breeds [17]. Thus, based on the RI for hot-blooded horses, ponies might have been falsely suspected of having a poorer prognosis or even being subjected to euthanasia. Blood LAC in healthy ponies have been determined only once in large population [18] but breed differences among ponies still remain unclear.

The commonly discussed differences between horses and ponies are glucose and triglyceride metabolism and cellular response to catecholamines [17, 19, 20], which may promote metabolic diseases [21, 22]. Ponies are less sensitive to insulin, so the rapid development of hypertriglyceridemia resulting from insulin resistance seems more likely [23, 24].

In the global literature, there are only a few reports dealing with haematology in the ponies. Thus, the aim of this study was to investigate the differences among blood parameters in ponies of different origin and height, represented by small (class A – up to 121 cm) ponies, Felin ponies and Polish Koniks and to compare theses values with commonly accepted RI for horses. Felin ponies and Polish Koniks are homogenous populations. The Polish Konik (*Equus cabalus gmelini* Ant.) is a primitive breed, descended directly from the wild Tarpans. They are small in stature (132–136 cm, so belong to height class C), have modest living requirements and high resistance to disease and environmental conditions [25]. Felin ponies represent a relatively new breed, specially dedicated for riding and competitive events for young riders. They originate from Arabians, Polish Koniks, Welsh ponies and Malopolski horses to whom they have the shortest genetic distance [26]. Malopolski horses were created from indigenous horses living in the eastern and north-western territories of Poland by crossings them with Thoroughbreds, Arabians and AngloArabians. Hence, while from genetic standpoint, Felin ponies may be treated as “hot-blooded” horses, according to the height (125–136 cm) they belong to class B or C.

## Results

Polish Koniks enrolled in the study were significantly younger than class A ponies (*p* = 0.001) and Felin ponies (*p* = 0.037). Moreover, there was significantly higher percentage of males in Polish Koniks than in the two remaining groups (*p* < 0.001) (Table 1). Therefore, this two demographic characteristics were included in statistical analysis as potential confounding factors.

**Table 1:** Demographic characteristics of the study population

Group	n	Height	Age [years]			
			Females		Males (no. of stallions)	
			n	Median, IQR (range)	n	Median, IQR (range)
Class A ponies (8 Shetland ponies, 3 Welsh ponies, 62 crossbreed ponies)	73	up to 121 cm	38	10, 8–10 (1–24)	35 (3)	14, 13–14 (1.5–31)
Felin ponies	28	125–136 cm	20	12, 5–19 (1.5–24)	8 (5)	10, 4–16 (2–29)
Polish Koniks	41	130–140 cm	38	7, 4–11 (2–16)	3 (3)	7, 10, 12

Hematological and biochemical parameters in class A ponies, Felin ponies and Polish Koniks are presented in Table 2 and Table 3.

**Table 2:** Hematological parameters in ponies class A, Felin ponies and Polish Koniks compared with so far published reference intervals for various types of horses (according to ^1^Rossdale & Partners, 2011 [5], ^2^Weiss & Wardrop, 2010 [7], ^3^Hinchcliff & Geor, 2013 [1])

Parameter^a^	Ponies class A (*n* = 73)	Felin ponies (*n* = 28)	Polish Koniks (*n* = 41)	General linear model *p*-value for the effect of				Reference intervals		
				groups	sex	age^c^	group & sex interaction	“hot-blooded” horses	“cold-blooded” horses	American Miniature Horses
RBC [T/L]	7.4 ± 1.0 (5.6–11.5)	7.9 ± 1.0 (6.3–10.7)	7.7 ± 0.9 (6.0–10.0)	0.201	0.609	< 0.001 −0.41 (− 0.58, − 0.24)	0.686	8.2 (6.2–10.2)^1^	5.5–9.5^2^	4.7–9.4^2^
HGB [mmol/L]	6.9 ± 0.9 (4.9–9.3)	7.1 ± 0.7 (6.0–8.4)	7.0 ± 0.6 (5.5–8.4)	0.466	0.883	0.927	0.673	8.4 (6.9–9.9)^1^	4.96–8.69^2^	5.52–10.12^2^
HCT [l/l]	0.35 ± 0.04 (0.30–0.50)	0.36 ± 0.03 (0.30–0.40)	0.35 ± 0.03 (0.30–0.40)	0.417	0.999	0.645	0.565	0.37 (0.31–0.43)^1^	0.24–0.44^2^	0.24–0.44^2^
MCV [fL]	46.9 ± 3.4 (36–55)	46.0 ± 3.8 (37–52)	46.0 ± 3.4 (39–51)	0.355	0.172	< 0.001 0.75 (0.61, 0.88)	0.126	46 (40.0–50.0)^1^		37–58^2^
MCH [fmol]	0.92 ± 0.07 (0.7–1.1)	0.91 ± 0.07 (0.7–1.0)	0.91 ± 0.07 (0.8–1.0)	0.336	0.170	< 0.001 0.66 (0.51, 0.80)	0.809	1.03 (0.94–1.18)^1^		0.81–1.43^2^
MCHC [mmol/L]	1.97 ± 0.06 (1.8–2.1)	1.97 ± 0.07 (1.8–2.1)	1.98 ± 0.06 (1.8–2.1)	0.788	0.490	0.015 −0.22 (−0.40, −0.04)	0.025	2.38 (2.07–2.40)^1^		2.17–2.48^2^
WBC [G/L]	7.1 ± 1.2 (4.5–10.7)	6.8 ± 1.4 (4.0–10.4)	7.5 ± 1.1 (5.5–10.3)	0.383	0.667	0.007 −0.24 (− 0.42, − 0.07)	0.197	7.5 (6.0–10.0)^1^	6.0–12.0^2^ 3.9–12.4^3^	5.0–14.97^2^ 10 ± 2.5^3^
GRA [G/L]	2.2 ± 0.7 (1.2–4.2)^A^	1.8 ± 0.5 (1.2–3.8)^A^	3.0 ± 0.7 (1.7–4.8)^B^	< 0.001	0.566	0.001 0.25 (0.10, 0.39)	0.774	4.4 (3.4–5.4)^1^	2.6–7.2^3^	3.7 ± 0.8^3^
LYM [G/L]	4.0 ± 1.0 (2.3–8.4)^A^	4.3 ± 1.1 (1.9–6.7)^A^	3.6 ± 0.9 (2.0–5.6)^B^	< 0.001	0.704	< 0.001 −0.56 (− 0.71, − 0.41)	0.177	2.6 (2.0–3.2)^1^	0.89–3.6^4^	5.9 ± 0.9^3^
MON [G/L]	0.9 ± 0.3 (0.3–1.5)	0.8 ± 0.2 (0.4–1.2)	0.9 ± 0.2 (0.6–1.5)	0.037^b^	0.426	< 0.001 0.36 (0.19, 0.52)	0.824	0.3 (0.2–0.4)^1^	0–0.62^4^	0.04 ± 0.08^3^
PLT [G/L]	277, 196–364 (95–900)^A^	435, 314–701 (181–900)^B^	403, 358–476 (116–900)^B^	< 0.001	0.981	0.453	0.452	156 (100–250)^1^		

*RBC* Number of red blood cells, *HGB* Haemoglobin concentration, *HCT* Haematocrit, *MCV* Mean corpuscular volume, *MCH* Mean corpuscular haemoglobin, *MCHC* Mean corpuscular haemoglobin concentration, *WBC* Number of white blood cells, *GRA* Number of granulocytes, *LYM* Number of lymphocytes, *MON* Number of monocytes, *PLT* Number of platelets

^a^ presented as arithmetic mean ± SD and range in parenthesis except for PLT which was non-normally distributed and presented as the median, interquartile range (IQR) and range in parenthesis

^b^ despite significant result of the omnibus test, pairwise comparisons were insignificant

^c^ standardized regression coefficient with 95% confidence interval (CI 95%) presented if significant

^A-C^ groups marked by different capital letters differ significantly in between-group pairwise comparisons at α = 0.05

**Table 3:** Blood biochemical parameters in ponies class A, Felin ponies and Polish Koniks compared with so far published reference intervals for various types of horses (according to ^1^Rossdale & Partners, 2011 [5], ^2^Hinchcliff & Geor, 2013 [1], ^3^Latson 2005 [9], Henderson 2013 [10])

Parameter^a^	Ponies class A (*n* = 73)	Felin ponies (*n* = 28)	Polish Koniks (*n* = 41)	General linear model *p*-value for the effect of				Reference intervals		
				groups	sex	age^b^	group & sex interaction	“hot-blooded” horses	“cold-blooded” horses	American Miniature Horses
ALP [U/l]	185 ± 81 (92–756)^AB^	149 ± 55 (72–281)^A^	211 ± 46 (126–358)^B^	0.036	0.327	0.104	0.424			
AST [U/l]	384 ± 83 (228–648)^A^	321 ± 50 (233–423)^B^	395 ± 65 (221–536)^A^	0.007	0.153	0.770	0.941	234 ± 163 (Clydesdales)^2^ 172 ± 28 (Shire)^2^	189 ± 33^2^	234 ± 163 (Clydesdales)^2^ 172 ± 28 (Shire)^2^
GGTP [U/l]	18, 15–21 (8–53)^A^	20, 18–25 (14–48)^A^	55, 45–82 (20–198)^B^	< 0.001	0.059	0.055	< 0.001	24.2 ± 6.0 (Shire)^2^	11 ± 4.4^2^	24.2 ± 6.0 (Shire)^2^
CPK [U/l]	473, 381–587 (171–1233)^A^	361, 277–408 (192–609)^B^	599, 459–769 (306–1137)^A^	0.001	0.037	0.855	0.059	56 ± 22 (Clydesdales)^2^ 58 ± 12 (Shire)^2^	273 ± 136^2^	56 ± 22 (Clydesdales)^2^ 58 ± 12 (Shire)^2^
TP [g/l]	66.8 ± 4.9 (56–75)	63.9 ± 5.6 (50–75)	66.1 ± 3.7 (57–75)	0.275	0.268	0.182	0.250	72 ± 6 (Clydesdales and Shire)^2^	66 ± 6^2^	72 ± 6 (Clydesdales and Shire)^2^
ALB [g/l]	35.6 ± 3.2 (26–41)^A^	37.8 ± 2.6 (34–46)^B^	36.1 ± 1.9 (32–41)^A^	0.003	0.116	0.635	0.684	36 ± 5 (Clydesdales)^2^ 28 ± 3 (Shire)^2^		36 ± 5 (Clydesdales)^2^ 28 ± 3 (Shire)^2^
Urea [mmol/L]	7.0 ± 1.4 (3.8–10.2)	7.0 ± 1.1 (5.1–9.0)	8.2 ± 1.5 (3.8–13.0)	0.812	0.034	0.776	0.019	5.2 ± 1.14 (Clydesdales)^2^	8.5 ± 1.4^2^	5.2 ± 1.14 (Clydesdales)^2^
Creatinine [μmol/L]	95.8 ± 16.9 (46.8–130.0)	98.8 ± 16.3 (61.9–137.0)	77.6 ± 17.1 (47.7–116.7)	0.357	0.004	0.983	0.051	151 ± 26.5 (Clydesdales)^2^	88.4 ± 17.7^2^	151 ± 26.5 (Clydesdales)^2^
TBIL [μmol/L]	16.9 ± 5.3 (5.3–32.3)^A^	20.6 ± 6.4 (11.1–37.6)^A^	12.3 ± 4.6 (4.4–28.7)^B^	0.001	< 0.001	0.216	0.015			
TG [mmol/L]	0.30 ± 0.15 (0.1–0.8)^A^	0.42 ± 0.20 (0.2–0.9)^B^	0.33 ± 0.12 (0.2–0.6)^AB^	0.029	0.504	0.002 −0.27 (−0.44, −0.10)	0.038			
Glucose [mmol/L]	5.4 ± 0.9 (3.9–8.4)^A^	5.1 ± 1.1 (3.6–9.1)^A^	3.8 ± 0.9 (2.2–6.3)^B^	< 0.001	0.270	0.809	0.138	4.21 ± 1.1 (Clydesdales)^2^	5.1 ± 0.7^2^	4.21 ± 1.1 (Clydesdales)^2^
Lactate [mmol/L]	1.1, 0.9–1.4 (0.1–2.7)^A^	0.1, 0.1–0.1 (0.1–0.9)^B^	0.1, 0.1–0.1 (0.1–2.6)^B^	< 0.001	0.428	0.157	0.534	234 ± 163 (Clydesdales)^2^ 172 ± 28 (Shire)^2^	189 ± 33^2^	234 ± 163 (Clydesdales)^2^ 172 ± 28 (Shire)^2^

*ALP* Alkaline phosphatase, *AST* Aspartate aminotransferase, *GGTP* Gamma-glutamyl transpeptidase, *CPK* Creatine kinase, *TP* Total protein concentration, *ALB* Albumin concentration, *TBIL* Total bilirubin, *TG* Triglycerides

^a^ presented as arithmetic mean ± SD and range in parenthesis except for CPK, GGTP, and lactate which were non-normally distributed and presented as the median, interquartile range (IQR) and range in parenthesis

^b^ standardized regression coefficient with 95% confidence interval (CI 95%) presented if significant

^A-B^ groups marked by different capital letters differ significantly in between-group pairwise comparisons at α = 0.05,

There were no differences in erythrogram parameters among the groups of ponies, but all values except HCT and MCV were slightly lower when compared to RI for non-thoroughbred (“hot-blooded”) horses [5]. WBC counts did not differ among ponies and were similar to the normal value for hot-blooded horses, but the proportion between GRA and LYM in ponies was inverted when compared to non-thoroughbred horses (the number of GRA was lower and LYM was higher) [5]. Both GRA and LYM differed significantly among groups of ponies. In Polish Koniks, the granulocyte count was significantly higher while the lymphocyte count was significantly lower than in class A ponies (*p* = 0.008 and *p* = 0.023, respectively) and Felin ponies (*p* < 0.001 and *p* = 0.009, respectively). PLT in all groups of ponies were much higher than those reported for hot-blooded horses [5], and differed significantly between class A ponies and both remaining groups - Felin ponies (*p* < 0.001) and Polish Koniks (*p* = 0.035).

The activities of blood enzymes (ALP, AST and GGTP) in ponies were generally higher than values accepted for hot-blooded horses [5] and differed also among the groups. The ALP activity was significantly lower in Felin ponies than in Polish Koniks (*p* = 0.002). The AST activity in Felin ponies was significantly lower than in class A ponies’ (*p* = 0.004) and Polish Koniks (*p* = 0.001). The GGTP activity was significantly higher in Polish Koniks than in class A ponies (*p* < 0.001) and Felin ponies (*p* < 0.001).

Total protein and albumin concentrations of ponies was similar to hot-blooded horses [5]. Albumin concentration differed significantly among ponies groups and was significantly higher in Felin ponies than in class A ponies (*p* = 0.002) and Polish Koniks (*p* = 0.038).

Blood urea was higher and creatinine was lower in ponies than in hot-blooded horses [5], however they did not differ significantly between the groups.

Liver parameters were generally lower in ponies than in hot-blooded horses [5]. Only TBIL in Felin ponies was the same as the normal value for hot-blooded horses and it was significantly higher than in other groups of ponies (*p* = 0.022). Moreover, TBIL was significantly lower in in Polish Koniks than in class A ponies (*p* = 0.012) and Felin ponies (*p* < 0.001). TG was low in all groups of ponies and significantly lower in class A ponies than in Felin ponies (*p* = 0.007).

Glucose concentration in class A ponies and Felin ponies was close to the upper RI limit for hot-blooded horses [5], and significantly higher than in Polish Koniks (*p* < 0.001 for both groups).

LAC was significantly higher in class A ponies’ (*p* < 0.001 when compared with other groups) and higher than normal value for hot-blooded horses [9, 10].

Sex proved to be a significant confounder in terms of CPK, urea, creatinine and TBIL. The former two were significantly higher in females, while the latter two in males.

Age was a significant confounder in terms of TG as well as of all hematological parameters except for HGB, HCT and PLT. Correlation with age was negative in terms of RBC, MCHC, WBC, LYM, and TG while positive with respect to MCV, MCH, GRA, and MON.

## Discussion

To the authors’ knowledge, current data regarding RI for haematological and blood biochemical parameters in ponies are limited. Additionally, it is still a matter for debate whether ponies should be classified as “hot-blooded” [8] or “cold-blooded” horses [7] from a haematological standpoint. Practitioners more often use RI for the former group.

It is commonly accepted that erythrogram values (RBC, HGB, HCT) are lower in cold-bloods than in hot-bloods. It has been stated that ponies’ haematocrit may be as low as 0.24 l/l, moreover, in American Miniature horses MCV, MCH and MCHC were higher [7]. WBC counts have been reported as slightly lower in cold-blooded than hot-blooded horses and neutrophil to lymphocyte ratios (N:L) were 1.0 in Arabians and Thoroughbreds, 1.7 in cold-blood horses and 0.67 in American Miniature horses [7].

Erythrogram values determined in the ponies enrolled in our study did not differ among groups and were much lower than accepted for adult Thoroughbreds and moderately lower than recommended for hot-blooded [5] and Hucul horses [27]. Hucul horses are small (132-145 cm high), so meet the general height criteria for ponies, but from genetic point are closer to hot-blooded horses. The differences between haematological parameters in Huculs and Felin ponies were quite surprising, due to ancestry, suggesting more similarities. It was also surprising that no pronounced differences were observed in HCT values between the ponies examined in our study and hot-blood horses. Similar haematological data have been reported for Noma and Kiso horses [4, 28], Shetland ponies [29], ponies in regular show jumping and eventing training [30] and Polish Koniks examined previously [31]. Interestingly, Bosnian ponies similar in size to Felin ponies and Polish Koniks had higher erythrogram values [32].

Leukogram values determined in our study were similar to values for hot-blooded horses but the N:L was below 1.0, confirming the previous findings in ponies [7]. However, Shawaf et al. [29] have shown that in Shetland ponies WBC was higher in summer than in winter, butregardless of season the values were higher than in our study. Also, the N:L ratio was above 1.0 in summer and winter [29]. In Noma horses WBC count was similar to the values determined in our study, but N:L ratio was above 1.0 [28]. It has been shown that granulocyte activity and the onset of inflammation differs between horses and Shetland ponies, being more robust in the latter [33]. Wilmink et al. [33] did not define the numbers of granulocytes, N:L ratios and WBCs, so we cannot compare our data with their findings. It might be postulated that the N:L ratio is one of breed related difference among ponies. However, it cannot be excluded that these differences were related to geographical region, as the cited studies were performed in Japan [28] and Saudi Arabia [29], not central Europe.

Erythrogram and leukogram values determined in our study were also related to the age of ponies, in the manner described recently in Spanish Purebreds [34]. Similarly as reported by Satue et al. [34], we observed that RBC decreased with age with compensatory increase of MCV and MCH. WBC also decreased with age, and although N:L ratio was not significantly increased in our study, neutrophil counts were higher and lymphocyte counts were lower, suggesting similar tendency. Vast similarities between the patterns of changes identified in our results and reported by Saute et al. [34] confirm their conclusion that age related changes pose the natural condition reflecting a decrease of the bone marrow response.

Haematological features, determined in our study pose an interesting breed and age related finding but of limited diagnostic importance. However, observed biochemical differences are diagnostically important.

One of the important findings in our study is a higher concentration of lactate in class A ponies, but not classes B and C represented by Felin ponies and Polish Koniks, respectively. This observation is important from the diagnostic standpoint, as blood and peritoneal LAC are commonly used as diagnostic and prognostic indicators in gastrointestinal and ischemic emergencies in horses. It has been reported that LAC measurement at admission and the changes over time discriminate between survivors and non-survivors and indicate the need for surgical intervention [11–13, 15–17, 35]. All the previously referenced studies involved large, but mixed breed populations with various conditions, so the values indicating guarded prognosis varied among reports. Mean blood lactate concentrations at admission for non-survivors were reported as 4.1 mmol/L with repeated measurements recommended [11]. Another studies suggested values below 2.1 [35] or above 9.548 mmol/L [13] for non-survivors and a cut-off value of < 6.0 mmol/L for survivors [13]. Delesalle et al., 2007 [15] stated an LAC value of 6.3 mmol/L for non-survivors, and recently an optimal decision tree has been proposed which identifies horses as non-survivors when the LAC at admission is ≥4.3 mmol/L [16]. Only in the last study ponies (27.3%) and Icelandic horses (31.8%) constituted the majority of the observed population, but breed differences in LAC measurements were not investigated. Ponies received more attention in Dunkel’s et al. study [17] where the mean LAC values for non-survivors were 4.1 and 3.1 mmol/L for ponies and horses, respectively, and did not differ significantly. However, the value for survivors was significantly higher in ponies (2.5 mmol/L) than in horses (1.2 mmol/L) and the authors concluded that ponies could be falsely suspected of having a surgical lesion or a poorer prognosis as they might present higher blood lactate concentrations. LAC concentrations in healthy ponies have been rarely determined. Recently, Dunkel et al. [18] published a study involving a large group of healthy ponies and determined that the mean LAC concentration was 0.7 (0.2–2.7) mmol/L and was lower than in full-sized horses: 0.9 (0.2–1.9) mmol/L. The authors concluded that lactate concentrations depended both on body condition and age [18]. Although the investigated population was large (101 animals), ponies were defined only on the basis of height (≤148 cm), and no data regarding breed or height classes were given. Our findings seem to substantially complement their study and provide additional clinically important information. In our study L-lactate concentrations, when compared to commonly accepted values for hot-blooded full-size horses < 0.7 mmol/L [9, 10] or 0.9 mmol/L [18] were high only in class A ponies’ (median of 1.1 mmol/L) but much lower in Felin ponies and Polish Koniks (median of 0.1 mmol/L in both) representing class B and C, respectively. The causes of different LAC concentration in ponies are unknown. However, LAC may be associated with either increased production or decreased clearance [12].

Several studies mentioned differences in hepatic functions between horses and ponies [17, 36]. Our study indicates that AST and GGTP in all groups of ponies are high and in Polish Koniks GGTP markedly exceeds the reference intervals for hot-blooded horses. In clinical conditions, elevation of AST and GGTP indicates hepatocellular injury [37]. The ponies in our study were clinically healthy and liver parameters were not high enough to indicate or even suggest hepatic disease, but physiological differences regarding the function of hepatocytes may be suspected. This hypothesis seems in line with the differences described in Noma and Kiso ponies [28]. In both Japanese breeds GGTP concentrations were lower than in the ponies examined in our study, but AST in Noma ponies was higher than in Kiso ponies [28] and very similar to the values measured in our study. However, in Shetland ponies in Saudi Arabia, GGTP and AST activities were comparably high to the values determined in our study only in the winter [29].

Difference regarding muscle enzymes also seem possible. CPK activity was surprisingly high in class A and Polish Koniks. Such high values have not been reported in Shetland ponies, Noma and Kiso ponies [28, 29]. In horses, high values of CPK and AST are indicators of muscle damage, and slight elevation are nonspecific [37]. CPK activities up to 10,000 U/l are considered small, nonspecific elevations [38], which may be associated with transport or exercise [37]. However, in performance horses much lower muscle enzyme activity occur after exercise [39, 40]. Ponies in our study were not transported and did not exercise. CPK activity never exceeded 1300 U/L, and AST activity was not high enough to suggest any muscle pathology. Thus, we postulate that high CPK values were related to muscle mass and composition, which were not examined in our study, but such differences in ponies have been reported in the literature [41]. This hypothesis seems in line also with observed in our study sex related differences in CPK activity, being higher in females.

Another important finding in our study was the low concentration of TG in all groups of ponies. Values were lower than referenced for hot-blooded horses and the lowest in the smallest class (class A ponies: 0.30 ± 0.15 mmol/l) and additionally, decreased with age. Metabolic differences between horses and ponies in glucose and triglyceride metabolism and tendencies to develop hyperlipemia in a negative energy balance are widely reported in ponies [17, 19, 20, 23, 42]. It is generally believed that healthy ponies may have higher TG levels [43] and thus, low TG concentrations are not of diagnostic relevance. Higher TG values 78.5 ± 11 mg/dl (0.89 ± 0.12 mmol/l) have been reported [44] but in more recent studies with ponies lower values predominate. Ono et al. [28] reported 37.2 ± 34.3 mg/dl (0.42 ± 0.39 mmol/L) for Noma horses and Takasu et al. [4] 16.4 ± 11.1 mg/L (0.19 ± 0.13 mmol/L) for Kiso horses. Even in pony mares in late gestation TG values as low as 11.3 ± 5.4 md/dl (0.13 ± 0.07 mmol/l) have been measured [45].

This is particularly important for practitioners because metabolic disturbances leading to hyperinsulinemia and equine metabolic syndrome (EMS) include a moderate elevation of TG concentrations [22, 23]. If ponies normally have low TG concentrations and practitioners use reference values for hot-blooded horses (reference interval from 0.2 to 1.2 mmol/L) [5], ponies actually having hypertriglyceridemia may be misdiagnosed, because ponies with mildly elevated TG, interpreted according to the reference values for hot-blooded horses, may in fact have markedly elevated TG. Thus, we postulate that the normal TG value for ponies should be reconsidered or at least treated with caution.

It is also noteworthy that in all groups of ponies examined by us, the concentrations of urea were higher and creatinine lower than recommended for full sized horses. Similar finding has been reported in American Miniature horses [1]. In Shetland, Noma and Kiso ponies creatinine concentrations were also low, but blood urea nitrogen (BUN) was lower than measured in our study [28, 29]. However, urea and creatinine concentrations appeared to be affected by sex rather than by breed.

Another finding in Polish Koniks is a significantly lower glucose concentration than in other groups of ponies and also lower than normal values reported for hot-blood horses [5]. This has also been reported in Shetland ponies, regardless of season [29], Noma and Kiso ponies [28] and mixed population of ponies [18]. However, it is similar to the value reported previously in Polish Koniks [25]. The differences between full-sized horses and ponies in glucose and insulin metabolism, oxidative capacity and response under sympathetic stimulation are widely reported in the literature [21–23, 36, 46]. Even though higher glucose concentrations, reflecting metabolic differences, have been clearly shown in ponies with gastrointestinal diseases [46], these differences are frequently not noticeable at rest but only during dynamic response testing [18, 21].

The main limitation of our study was the number of ponies in examined groups, too small to establish reliable reference intervals for groups. It should also be mentioned that differences in haematological and biochemical values may be associated with the method of the test or even equipment. The methods used in our study are routine in other laboratories, except hand-held lactate and glucose analysers, that are used in field practice. Accusport analyser used in this study has been evaluated in horses and successfully used in other studies. It has been shown that Accusport is reliable and accurately measured plasma lactate concentration in horses so that they are comparable to values from other analysers [47, 48]. The differences in age and sex distribution between groups were controlled in the statistical analysis so it is unlikely that they negatively affected the results obtained.

## Conclusions

In conclusion, our study proved breed related differences in haematological and blood biochemical parameters in the ponies. In our opinion, normal values for ponies should vary at least with the height classes. Special attention should be paid on the interpretation of blood LAC and TG values, especially in cases where ponies are being evaluated for clinical disease.

## Methods

The study involved 142 riding ponies, divided into 3 groups (Table 1). The first group (*n* = 73) consisted of animals classified on the basis of height as class A ponies (up to 121 cm) and included 8 Shetland ponies, 3 Welsh ponies, 62 crossbreed ponies. Both genders were represented and ponies were from 2 to 31 years of age. The second group was composed of Felin ponies (*n* = 28) aged 2 to 29 years, both genders. The third group comprised Polish Konik horses (*n* = 41) aged 2 to 16 years, both genders.

The animals were stabled in various horse facilities in central Poland and were managed in a similar manner, including standard diet and similar daily activity. All owners gave the informed consent for the procedure. All horses were dewormed and vaccinated according to standard protocols in the stable. None of these procedures had taken place less than 3 weeks before the blood collection and no veterinary medicinal products have been administered to the ponies during this period. All samples were obtained during routine veterinary procedures (health check), performed in August, under similar weather conditions. All animals were examined in the morning, when they were in stalls, handled by their keepers to minimalize stress. Immediately after the procedure, the animals returned to their routine activities. The standard clinical examination revealed no clinical signs of disease and no abnormalities were reported during 1 month before examination. All mares were in anoestrus phase.

All blood samples were obtained by the jugular venipuncture using a BD Vacutainer system into K2-EDTA tubes for haematological tests and serum analyses. EDTA blood samples were kept at + 4 °C and they were examined within 5 h of collection for the following haematological parameters: white blood cell count (WBC), granulocyte count (GRA), lymphocyte count (LYM), monocyte count (MON), haematocrit (HCT), haemoglobin concentration (HGB), red blood cell count (RBC), mean corpuscular volume (MCV), mean corpuscular haemoglobin (MCH), mean corpuscular haemoglobin concentration (MCHC) and the total number of platelets (PLT) in an automated analyser calibrated for equine species (ABC Vet, Horiba ABX).

The tubes with no anticoagulant were centrifuged at 4380 g for 5 min, serum was aspirated and analysed. None of the collected blood samples had visual signs of haemolysis. Clinical biochemistry analyses included the activity of alkaline phosphatase (ALP), aspartate aminotransferase (AST), gamma-glutamyl transpeptidase (GGTP), and creatine kinase (CPK); serum concentration of albumin (ALB), total bilirubin (TBIL), triglycerides (TG), urea and creatinine. They were performed using an automated clinical biochemistry analyser (Miura One, ISE. S.r.l., Italy). Total protein (TP) concentration was measured by refractometer technique (Reichert Rhino Vet 360). For all measurements Pointe Scientific (USA) reagents, standards, calibrators and controls were used. Blood LAC and glucose concentrations were determined immediately after blood collection using the Accutrend Plus (Roche Diagnostics) and OptiumXido (Abbott Diabetes Care), respectively.

Statistical analysis was performed in TIBCO Statistica 13.3 (TIBCO Software Inc.). Categorical variables were presented as the count and percentage in the group and compared between groups using the Pearson’s chi-square test. Numerical variables were given as the arithmetic mean and standard deviation (±SD) or the median and interquartile range (IQR) unless a variable was normally distributed. Range was presented in all cases. Normality of distribution was assessed on the basis of histograms and the Shapiro-Wilk W test, and non-normally distributed variables (i.e. age, PLT, GGTP, CPK, and lactate) were transformed with logarithmical (natural logarithm) or Box-Cox transformation. Blood parameters compared between groups using the general linear model (GLM) which included sex and age as potential confounders as well as the interaction between the group and sex to control for unbalanced distribution of males and females across the groups. If GLM yielded significant result pairwise between-group comparisons were performed with the Tukey’s honestly significant difference post-hoc test for groups of unequal size. All tests were two-sided and the significance level (α) was set at 0.05.

## Data Availability

The datasets used and/or analysed during the current study are available from the corresponding author on reasonable request.

## Abbreviations

ALB: Albumin
ALP: Alkaline phosphatase,
AST: Aspartate aminotransferase,
CPK: Creatine kinase,
GGTP: Gamma-glutamyl transpeptidase,
GLM: General linear model.
GRA: Granulocyte count,
HCT: Haematocrit,
HGB: Haemoglobin concentration,
IQR: Interquartile range,
LAC: L-lactate
LYM: Lymphocyte count,
MCH: Mean corpuscular haemoglobin,
MCHC: Mean corpuscular haemoglobin concentration,
MCV: Mean corpuscular volume,
MON: Monocyte count,
N:L: Neutrophil to lymphocyte ratio
PLT: Platelet count,
RBC: Red blood cell count,
SD: Standard deviation,
TBIL: Total bilirubin,
TG: Triglycerides,
TP: Total protein,
WBC: White blood cell count.
