# Correction to: Variations in haematological and biochemical parameters in healthy ponies

**DOI:** 10.1186/s12917-021-02859-0

**Published:** 2021-04-02

**Authors:** Olga Witkowska-Piłaszewicz, Anna Cywińska, Katarzyna Michlik-Połczyńska, Michał Czopowicz, Katarzyna Strzelec, Anna Biazik, Marta Parzeniecka-Jaworska, Mark Crisman, Lucjan Witkowski

**Affiliations:** 1 Department of Pathology and Veterinary Diagnostics, Institute of Veterinary Medicine, Warsaw University of Life Sciences – SGGW, Nowoursynowska str. 159c, 02-787 Warsaw, Poland; 2 Faculty of Biological and Veterinary Sciences, Nicolaus Copernicus University in Toruń, Toruń, Poland; 3 Department of Internal Diseases and Veterinary Diagnostics, Faculty of Veterinary Medicine and Animal Science, Poznan University of Life Sciences, Poznań, Poland; 4 Division of Veterinary Epidemiology and Economics, Institute of Veterinary Medicine, Warsaw University of Life Sciences – SGGW, Warsaw, Poland; 5 Department of Horse Breeding and Use, University of Life Sciences in Lublin, Lublin, Poland; 6 Department of Large Animal Clinical Science, Virginia Maryland College of Veterinary Medicine, Virginia Tech, Blacksburg, USA

**Correction to: BMC Vet Res 17, 38 (2021)**

**https://doi.org/10.1186/s12917-020-02741-5**

The original article [1] contained an error whereby all authors’ names were mistakenly inverted. This error has since been corrected.
